# A Case Report of a Spontaneous Venous Aneurysm Present for 25 Years

**DOI:** 10.7759/cureus.96898

**Published:** 2025-11-15

**Authors:** Arya Singh, Dave Harnanan, Yardesh Singh, Vijay Naraynsingh

**Affiliations:** 1 Medicine, Royal College of Surgeons in Ireland, Dublin, IRL; 2 Clinical Surgical Sciences, The University of the West Indies, St. Augustine, TTO; 3 Surgery, Medical Associates Hospital, St. Joseph, TTO

**Keywords:** distal arm, general and vascular surgery, spontaneous venous aneurysm, vascular abnormality, venous aneurysm

## Abstract

Venous aneurysms (VAs) are uncommon vascular abnormalities characterized by a focal dilation of a vein, wherein the vessel is markedly enlarged compared to its original size. VAs can be classified as either superficial or deep. A 60-year-old female patient presented with a 25-year history of a spontaneous VA on her wrist. Throughout this duration, the patient experienced no pain, only mild discomfort. The aneurysm was superficial, subcutaneous, and diagnosed through clinical examination. Surgical ligation and excision were performed under local anesthesia. Swellings at this site on the wrist can be due to a ganglion, arteriovenous (A-V) fistula, implantation dermoid, or sebaceous cyst; however, this uncommon VA had distinctive clinical features. VAs are often diagnosed using duplex ultrasound, magnetic resonance venography, and blood tests. Definitive treatment involves surgical ligation and excision of the aneurysm.

## Introduction

A venous aneurysm (VA) is the dilation of a vein, specifically when it is at minimum 1.5 times larger than the adjacent nondilated vein [1]. VAs are classified as either superficial or deep and can be found at various sites, such as the intra- and extra-cranial veins, the extremities, the superior vena cava, and the common iliac and spleno-portal systems [2]. VAs are usually preceded by trauma and are at risk of thromboembolism [3]. A spontaneous VA refers to one that occurs without an explanation. VAs are typically diagnosed using duplex scans and treated surgically through ligation and excision.

## Case presentation

**Figure 1:**
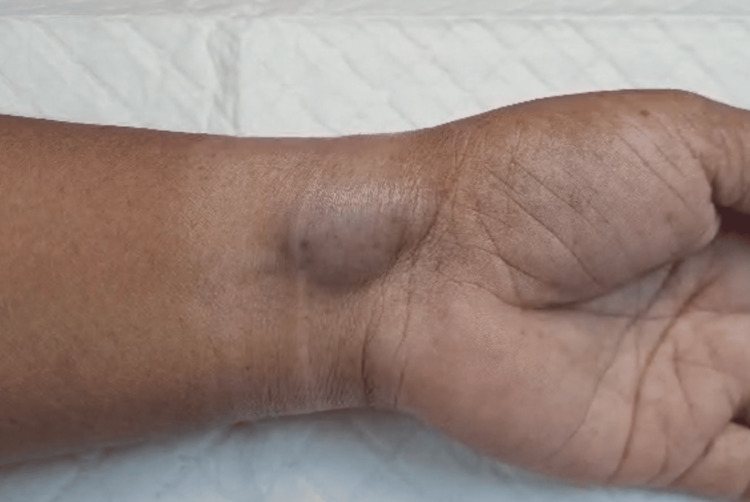
The inflated venous aneurysm when the wrist was below the level of the heart.

**Figure 2:**
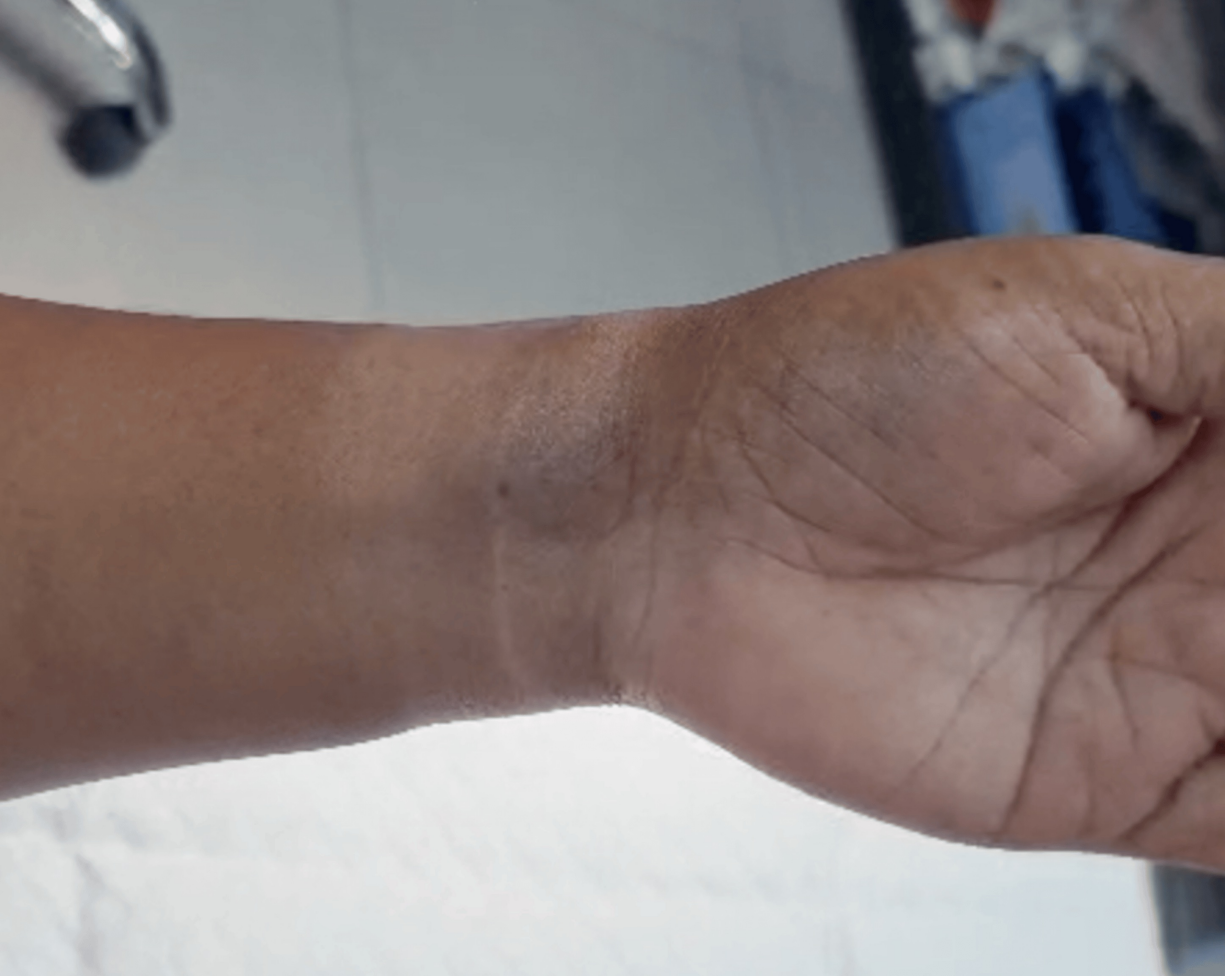
The collapsed venous aneurysm when the wrist was elevated above the level of the heart.

In August 2025, a 60-year-old female patient with no known illnesses and no medications had noticed a bulging on her wrist 25 years ago. It increased in size very slowly; there was no pain, just mild discomfort at the site. The patient had no preceding trauma. Upon examination, there was a 3 x 2.5 cm mass on the anterior aspect of the wrist. It had a slight purple discoloration and was soft and compressible at the site. The patient had a full range of motion of the wrist but experienced slight discomfort. There was no bruit, thrill, or pulsation. Figure 1 depicts the vein in its usual distended state and filled with blood when resting below heart level. However, when the patient's wrist was raised above heart level, the vein emptied (Figure 2). Similarly, when pressure was applied externally to the distended vein, it was emptied, and upon removal of the pressure, the blood returned slowly to the abnormal area, causing the skin to raise once more.

Surgical treatment included ligation and excision of the VA under local anesthesia. A transverse incision made over the raised area exposed the subcutaneous aneurysm. The arm was then raised above heart level to empty the expanded vein to facilitate dissection without rupturing the VA. The now-deflated vein was ligated and excised. 

Histology confirmed VA with no vasculitis or thrombosis. She was discharged immediately post-operation and was asymptomatic two weeks later.

## Discussion

The first description of VA was from an autopsy by Osler in 1913 [4]. Furthermore, VAs are classified as either superficial or deep based on the venous system involved [1]. In the arm, superficial VAs arise in the brachiocephalic and basilic veins, while deep VAs develop deep to the fascia of the arm [1]. VAs are often misdiagnosed as soft tissue masses [2,3]. They are more likely to occur in patients with high body mass index.

Most cases of VAs are preceded by trauma and are usually asymptomatic [1,5]. Patients with VAs may present with edema, swelling, and pain [1]. In our case, the patient had a 25-year history of VA. She experienced only mild discomfort due to the increasing size of the aneurysm and no other symptoms. This 25-year history suggests that there were no complications such as vasculitis and thromboembolism, the risk of which increases due to VAs [6]. Presentation plays a key role in characterization, as thromboembolism is more likely to occur in deep VAs as opposed to superficial ones [3].

To investigate, tests such as a duplex scan followed by a CT scan may be useful. Venous duplex imaging assesses the size, morphology, and presence of a thrombus in the extremities, while the CT scan is performed to assess deep VAs. Other investigations include magnetic resonance venography and ultrasound [7,8]. Although ganglion cysts, dermoid cysts, hemangiomas, lipomas, neurofibromas, aneurysms, and arteriovenous (A-V) fistulas enter the differential diagnosis, they do not disappear so quickly and completely upon simple elevation of the limb above cardiac level. If there is doubt about the diagnosis, an ultrasound may be very useful. On occasion, as in the case described, the patient's diagnosis can be made solely based on clinical examination.

VAs can occur secondary to trauma, inflammation, degenerative changes in the vessel wall, or increased pressure within the vascular system [9]. Schatz and Fine hypothesized that an important factor in the pathogenesis of VAs is endophlebohypertrophy [10]. This concept suggests that with increased venous outflow, hypertrophy of the vein wall occurs, leading to dilation and sclerosis. However, deep VAs are caused by intrinsic wall weakness and are not related to blood flow.

Treatment for VAs is usually surgical, but can also include cyanoacrylate or thrombin injection [11,12]. Fusiform aneurysms are treated with resection and end-to-end anastomosis or interposition grafts, while bypass or ligation of proximal and distal veins is also an option. Superficial aneurysms can be treated by ligation of the afferent and efferent veins, as seen in our case. In this case, a transverse incision was made on the patient’s wrist along Langer’s lines. This approach leads to a more aesthetic cosmetic result and does not affect the surgical procedure. Another technique used was the elevation of the arm above the level of the heart to empty the vein, aiding dissection. This maneuver helped prevent rupture of the already thin-walled vein; the dissection of the vein was easier as the collapsed vein facilitated dissection around the sides and deep surface without rupture.

Most aneurysms, whether deep or superficial, can be surgically treated with simple ligation and excision, but not all require surgical intervention. Similar to our case, McKesey et al. described a case involving a 59-year-old patient with a spontaneous VA on the wrist who avoided surgical intervention due to the absence of complications [1]. It is deemed safe to follow asymptomatic patients over time and to intervene only when the aneurysm becomes large or symptomatic [13]. Our case remained untreated for 25 years and was largely asymptomatic; the patient eventually wished for surgery only due to the very slow, continued increase in size and intermittent mild discomfort.

## Conclusions

VA is an uncommon vascular abnormality characterized by the abnormal dilation of a vein. It can be asymptomatic or present with edema, pain, and swelling, and may lead to thromboembolism. Patients with a long history of VA, such as in this case, are unlikely to be symptomatic or experience any complications. Investigations include venous duplex imaging and CT scans or MRI. Similar to the case mentioned, some instances do not require extensive investigations, as clinical assessment is sufficient. Furthermore, treatment is usually performed surgically through ligation and excision, as in our case, but it is not always required.
